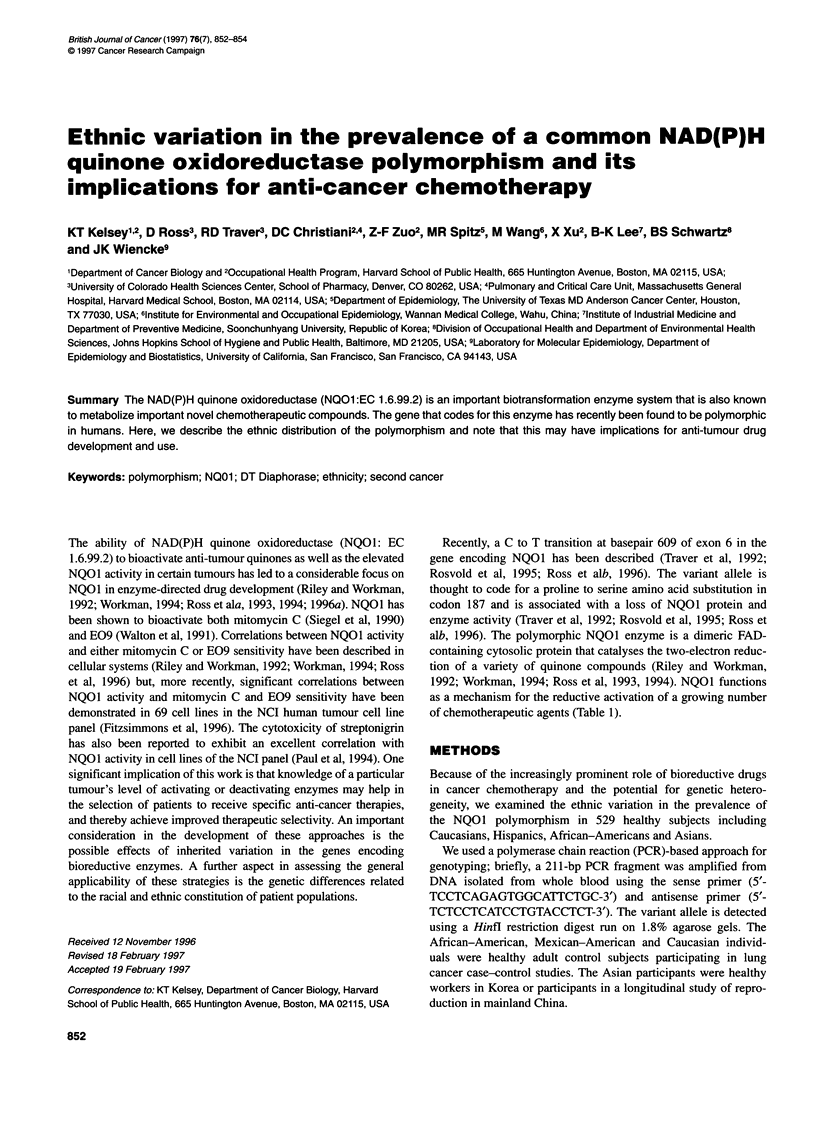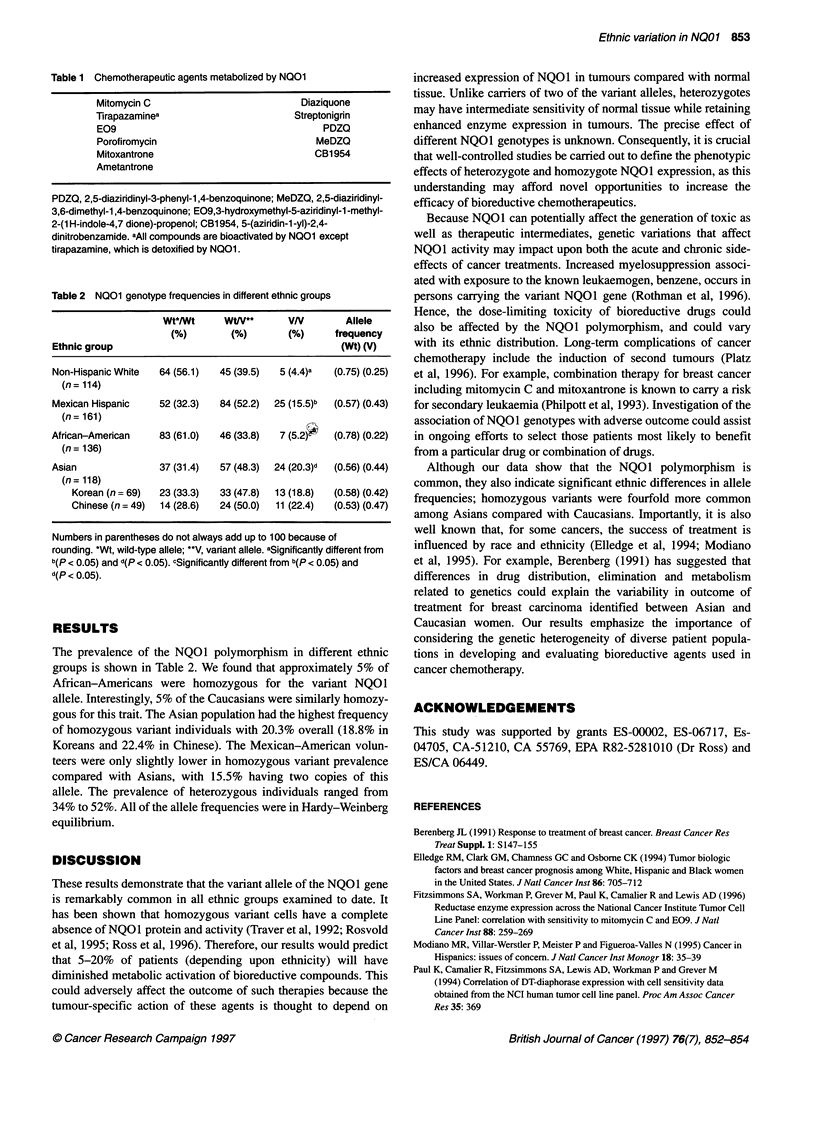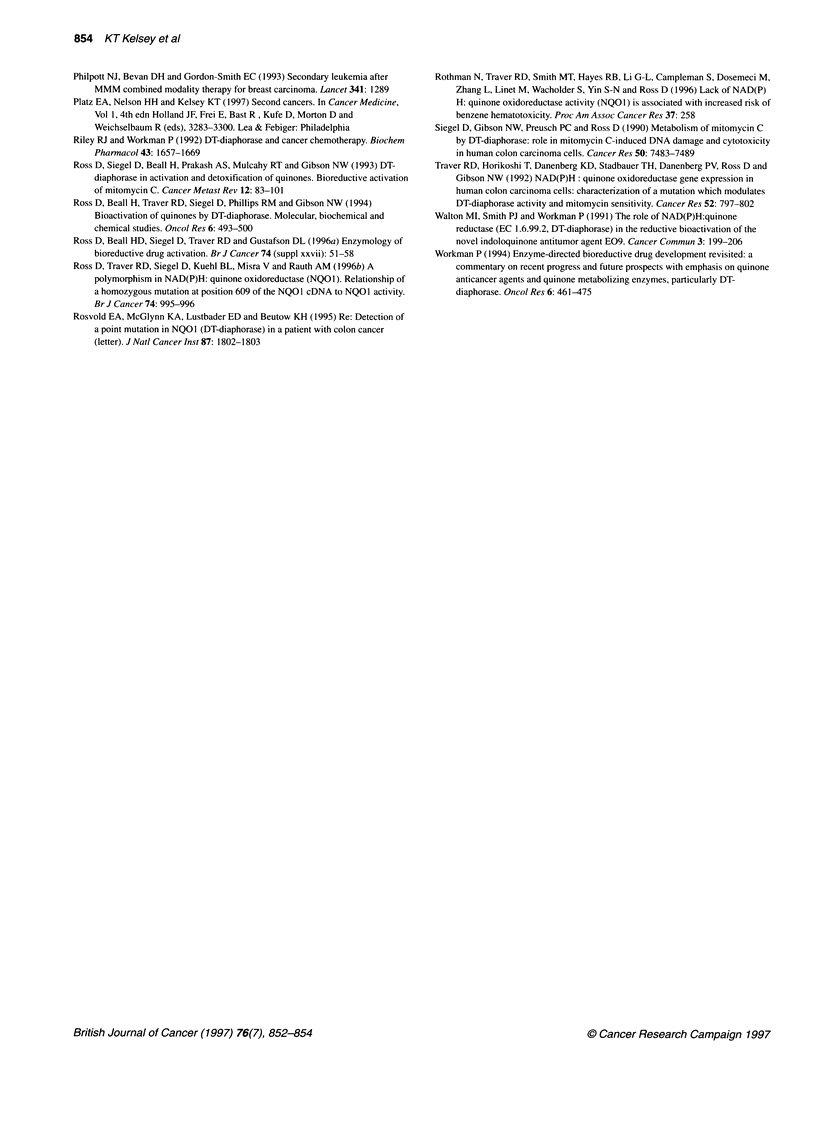# Ethnic variation in the prevalence of a common NAD(P)H quinone oxidoreductase polymorphism and its implications for anti-cancer chemotherapy.

**DOI:** 10.1038/bjc.1997.474

**Published:** 1997

**Authors:** K. T. Kelsey, D. Ross, R. D. Traver, D. C. Christiani, Z. F. Zuo, M. R. Spitz, M. Wang, X. Xu, B. K. Lee, B. S. Schwartz, J. K. Wiencke

**Affiliations:** Department of Cancer Biology, Harvard School of Public Health, Boston, MA 02115, USA.

## Abstract

The NAD(P)H quinone oxidoreductase (NQO1:EC 1.6.99.2) is an important biotransformation enzyme system that is also known to metabolize important novel chemotherapeutic compounds. The gene that codes for this enzyme has recently been found to be polymorphic in humans. Here, we describe the ethnic distribution of the polymorphism and note that this may have implications for anti-tumour drug development and use.


British Journal of Cancer (1997) 76(7), 852–854
© 1997 Cancer Research Campaign

### Ethnic variation in the prevalence of a common NAD(P)H quinone oxidoreductase polymorphism and its implications for anti-cancer chemotherapy

**KT Kelsey[1,2], D Ross[3], RD Traver[3], DC Christiani[2,4], Z-F Zuo[2], MR Spitz[5], M Wang[6], X Xu[2], B-K Lee[7], BS Schwartz[8] and JK Wiencke[9]**

[1]Department of Cancer Biology and [2]Occupational Health Program, Harvard School of Public Health, 665 Huntington Avenue, Boston, MA 02115, USA; [3]University of Colorado Health Sciences Center, School of Pharmacy, Denver, CO 80262, USA; [4]Pulmonary and Critical Care Unit, Massachusetts General Hospital, Harvard Medical School, Boston, MA 02114, USA; [5]Department of Epidemiology, The University of Texas MD Anderson Cancer Center, Houston, TX 77030, USA; [6]Institute for Environmental and Occupational Epidemiology, Wannan Medical College, Wahu, China; [7]Institute of Industrial Medicine and Department of Preventive Medicine, Soonchunhyang University, Republic of Korea; [8]Division of Occupational Health and Department of Environmental Health Sciences, Johns Hopkins School of Hygiene and Public Health, Baltimore, MD 21205, USA; [9]Laboratory for Molecular Epidemiology, Department of Epidemiology and Biostatistics, University of California, San Francisco, San Francisco, CA 94143, USA

**Summary** The NAD(P)H quinone oxidoreductase (NQO1:EC 1.6.99.2) is an important biotransformation enzyme system that is also known to metabolize important novel chemotherapeutic compounds. The gene that codes for this enzyme has recently been found to be polymorphic in humans. Here, we describe the ethnic distribution of the polymorphism and note that this may have implications for anti-tumour drug development and use.

**Keywords:** polymorphism; NQ01; DT Diaphorase; ethnicity; second cancer

The ability of NAD(P)H quinone oxidoreductase (NQO1: EC 1.6.99.2) to bioactivate anti-tumour quinones as well as the elevated NQO1 activity in certain tumours has led to a considerable focus on NQO1 in enzyme-directed drug development (Riley and Workman, 1992; Workman, 1994; Ross et al*a*, 1993, 1994; 1996*a*). NQO1 has been shown to bioactivate both mitomycin C (Siegel et al, 1990) and EO9 (Walton et al, 1991). Correlations between NQO1 activity and either mitomycin C or EO9 sensitivity have been described in cellular systems (Riley and Workman, 1992; Workman, 1994; Ross et al, 1996) but, more recently, significant correlations between NQO1 activity and mitomycin C and EO9 sensitivity have been demonstrated in 69 cell lines in the NCI human tumour cell line panel (Fitzsimmons et al, 1996). The cytotoxicity of streptonigrin has also been reported to exhibit an excellent correlation with NQO1 activity in cell lines of the NCI panel (Paul et al, 1994). One significant implication of this work is that knowledge of a particular tumour's level of activating or deactivating enzymes may help in the selection of patients to receive specific anti-cancer therapies, and thereby achieve improved therapeutic selectivity. An important consideration in the development of these approaches is the possible effects of inherited variation in the genes encoding bioreductive enzymes. A further aspect in assessing the general applicability of these strategies is the genetic differences related to the racial and ethnic constitution of patient populations.

*Received 12 November 1996*
*Revised 18 February 1997*
*Accepted 19 February 1997*

*Correspondence to:* KT Kelsey, Department of Cancer Biology, Harvard School of Public Health, 665 Huntington Avenue, Boston, MA 02115, USA

Recently, a C to T transition at basepair 609 of exon 6 in the gene encoding NQO1 has been described (Traver et al, 1992; Rosvold et al, 1995; Ross et al*b*, 1996). The variant allele is thought to code for a proline to serine amino acid substitution in codon 187 and is associated with a loss of NQO1 protein and enzyme activity (Traver et al, 1992; Rosvold et al, 1995; Ross et al*b*, 1996). The polymorphic NQO1 enzyme is a dimeric FAD-containing cytosolic protein that catalyses the two-electron reduction of a variety of quinone compounds (Riley and Workman, 1992; Workman, 1994; Ross et al, 1993, 1994). NQO1 functions as a mechanism for the reductive activation of a growing number of chemotherapeutic agents (Table 1).

### METHODS

Because of the increasingly prominent role of bioreductive drugs in cancer chemotherapy and the potential for genetic heterogeneity, we examined the ethnic variation in the prevalence of the NQO1 polymorphism in 529 healthy subjects including Caucasians, Hispanics, African–Americans and Asians.

We used a polymerase chain reaction (PCR)-based approach for genotyping; briefly, a 211-bp PCR fragment was amplified from DNA isolated from whole blood using the sense primer (5′-TCCTCAGAGTGGCATTCTGC-3′) and antisense primer (5′-TCTCCTCATCCTGTACCTCT-3′). The variant allele is detected using a *Hin*fI restriction digest run on 1.8% agarose gels. The African–American, Mexican–American and Caucasian individuals were healthy adult control subjects participating in lung cancer case–control studies. The Asian participants were healthy workers in Korea or participants in a longitudinal study of reproduction in mainland China.

**Table 1** Chemotherapeutic agents metabolized by NQO1

| | |
|---|---|
| Mitomycin C | Diaziquone |
| Tirapazamine[a] | Streptonigrin |
| EO9 | PDZQ |
| Porofiromycin | MeDZQ |
| Mitoxantrone | CB1954 |
| Ametantrone | |

PDZQ, 2,5-diaziridinyl-3-phenyl-1,4-benzoquinone; MeDZQ, 2,5-diaziridinyl-3,6-dimethyl-1,4-benzoquinone; EO9,3-hydroxymethyl-5-aziridinyl-1-methyl-2-(1H-indole-4,7 dione)-propenol; CB1954, 5-(aziridin-1-yl)-2,4-dinitrobenzamide. [a]All compounds are bioactivated by NQO1 except tirapazamine, which is detoxified by NQO1.

**Table 2** NQO1 genotype frequencies in different ethnic groups

| Ethnic group | Wt*/Wt (%) | Wt/V** (%) | V/V (%) | Allele frequency (Wt) (V) |
|---|---|---|---|---|
| Non-Hispanic White ($n$ = 114) | 64 (56.1) | 45 (39.5) | 5 (4.4)[a] | (0.75) (0.25) |
| Mexican Hispanic ($n$ = 161) | 52 (32.3) | 84 (52.2) | 25 (15.5)[b] | (0.57) (0.43) |
| African–American ($n$ = 136) | 83 (61.0) | 46 (33.8) | 7 (5.2)[c] | (0.78) (0.22) |
| Asian ($n$ = 118) | 37 (31.4) | 57 (48.3) | 24 (20.3)[d] | (0.56) (0.44) |
| Korean ($n$ = 69) | 23 (33.3) | 33 (47.8) | 13 (18.8) | (0.58) (0.42) |
| Chinese ($n$ = 49) | 14 (28.6) | 24 (50.0) | 11 (22.4) | (0.53) (0.47) |

Numbers in parentheses do not always add up to 100 because of rounding. *Wt, wild-type allele; **V, variant allele. [a]Significantly different from [b]($P < 0.05$) and [d]($P < 0.05$). [c]Significantly different from [b]($P < 0.05$) and [d]($P < 0.05$).

### RESULTS

The prevalence of the NQO1 polymorphism in different ethnic groups is shown in Table 2. We found that approximately 5% of African–Americans were homozygous for the variant NQO1 allele. Interestingly, 5% of the Caucasians were similarly homozygous for this trait. The Asian population had the highest frequency of homozygous variant individuals with 20.3% overall (18.8% in Koreans and 22.4% in Chinese). The Mexican–American volunteers were only slightly lower in homozygous variant prevalence compared with Asians, with 15.5% having two copies of this allele. The prevalence of heterozygous individuals ranged from 34% to 52%. All of the allele frequencies were in Hardy–Weinberg equilibrium.

### DISCUSSION

These results demonstrate that the variant allele of the NQO1 gene is remarkably common in all ethnic groups examined to date. It has been shown that homozygous variant cells have a complete absence of NQO1 protein and activity (Traver et al, 1992; Rosvold et al, 1995; Ross et al, 1996). Therefore, our results would predict that 5–20% of patients (depending upon ethnicity) will have diminished metabolic activation of bioreductive compounds. This could adversely affect the outcome of such therapies because the tumour-specific action of these agents is thought to depend on increased expression of NQO1 in tumours compared with normal tissue. Unlike carriers of two of the variant alleles, heterozygotes may have intermediate sensitivity of normal tissue while retaining enhanced enzyme expression in tumours. The precise effect of different NQO1 genotypes is unknown. Consequently, it is crucial that well-controlled studies be carried out to define the phenotypic effects of heterozygote and homozygote NQO1 expression, as this understanding may afford novel opportunities to increase the efficacy of bioreductive chemotherapeutics.

Because NQO1 can potentially affect the generation of toxic as well as therapeutic intermediates, genetic variations that affect NQO1 activity may impact upon both the acute and chronic side-effects of cancer treatments. Increased myelosuppression associated with exposure to the known leukaemogen, benzene, occurs in persons carrying the variant NQO1 gene (Rothman et al, 1996). Hence, the dose-limiting toxicity of bioreductive drugs could also be affected by the NQO1 polymorphism, and could vary with its ethnic distribution. Long-term complications of cancer chemotherapy include the induction of second tumours (Platz et al, 1996). For example, combination therapy for breast cancer including mitomycin C and mitoxantrone is known to carry a risk for secondary leukaemia (Philpott et al, 1993). Investigation of the association of NQO1 genotypes with adverse outcome could assist in ongoing efforts to select those patients most likely to benefit from a particular drug or combination of drugs.

Although our data show that the NQO1 polymorphism is common, they also indicate significant ethnic differences in allele frequencies; homozygous variants were fourfold more common among Asians compared with Caucasians. Importantly, it is also well known that, for some cancers, the success of treatment is influenced by race and ethnicity (Elledge et al, 1994; Modiano et al, 1995). For example, Berenberg (1991) has suggested that differences in drug distribution, elimination and metabolism related to genetics could explain the variability in outcome of treatment for breast carcinoma identified between Asian and Caucasian women. Our results emphasize the importance of considering the genetic heterogeneity of diverse patient populations in developing and evaluating bioreductive agents used in cancer chemotherapy.

### ACKNOWLEDGEMENTS

This study was supported by grants ES-00002, ES-06717, Es-04705, CA-51210, CA 55769, EPA R82-5281010 (Dr Ross) and ES/CA 06449.

### REFERENCES

Berenberg JL (1991) Response to treatment of breast cancer. *Breast Cancer Res Treat* **Suppl. 1**: S147–155

Elledge RM, Clark GM, Chamness GC and Osborne CK (1994) Tumor biologic factors and breast cancer prognosis among White, Hispanic and Black women in the United States. *J Natl Cancer Inst* **86**: 705–712

Fitzsimmons SA, Workman P, Grever M, Paul K, Camalier R and Lewis AD (1996) Reductase enzyme expression across the National Cancer Institute Tumor Cell Line Panel: correlation with sensitivity to mitomycin C and EO9. *J Natl Cancer Inst* **88**: 259–269

Modiano MR, Villar-Werstler P, Meister P and Figueroa-Valles N (1995) Cancer in Hispanics: issues of concern. *J Natl Cancer Inst Monogr* **18**: 35–39

Paul K, Camalier R, Fitzsimmons SA, Lewis AD, Workman P and Grever M (1994) Correlation of DT-diaphorase expression with cell sensitivity data obtained from the NCI human tumor cell line panel. *Proc Am Assoc Cancer Res* **35**: 369

© *Cancer Research Campaign 1997*

Philpott NJ, Bevan DH and Gordon-Smith EC (1993) Secondary leukemia after MMM combined modality therapy for breast carcinoma. *Lancet* **341**: 1289

Platz EA, Nelson HH and Kelsey KT (1997) Second cancers. In *Cancer Medicine*, Vol 1, 4th edn Holland JF, Frei E, Bast R , Kufe D, Morton D and Weichselbaum R (eds), 3283–3300. Lea & Febiger: Philadelphia

Riley RJ and Workman P (1992) DT-diaphorase and cancer chemotherapy. *Biochem Pharmacol* **43**: 1657–1669

Ross D, Siegel D, Beall H, Prakash AS, Mulcahy RT and Gibson NW (1993) DT-diaphorase in activation and detoxification of quinones. Bioreductive activation of mitomycin C. *Cancer Metast Rev* **12**: 83–101

Ross D, Beall H, Traver RD, Siegel D, Phillips RM and Gibson NW (1994) Bioactivation of quinones by DT-diaphorase. Molecular, biochemical and chemical studies. *Oncol Res* **6**: 493–500

Ross D, Beall HD, Siegel D, Traver RD and Gustafson DL (1996*a*) Enzymology of bioreductive drug activation. *Br J Cancer* **74** (suppl xxvii): 51–58

Ross D, Traver RD, Siegel D, Kuehl BL, Misra V and Rauth AM (1996*b*) A polymorphism in NAD(P)H: quinone oxidoreductase (NQO1). Relationship of a homozygous mutation at position 609 of the NQO1 cDNA to NQO1 activity. *Br J Cancer* **74**: 995–996

Rosvold EA, McGlynn KA, Lustbader ED and Beutow KH (1995) Re: Detection of a point mutation in NQO1 (DT-diaphorase) in a patient with colon cancer (letter). *J Natl Cancer Inst* **87**: 1802–1803

Rothman N, Traver RD, Smith MT, Hayes RB, Li G-L, Campleman S, Dosemeci M, Zhang L, Linet M, Wacholder S, Yin S-N and Ross D (1996) Lack of NAD(P)H: quinone oxidoreductase activity (NQO1) is associated with increased risk of benzene hematotoxicity. *Proc Am Assoc Cancer Res* **37**: 258

Siegel D, Gibson NW, Preusch PC and Ross D (1990) Metabolism of mitomycin C by DT-diaphorase: role in mitomycin C-induced DNA damage and cytotoxicity in human colon carcinoma cells. *Cancer Res* **50**: 7483–7489

Traver RD, Horikoshi T, Danenberg KD, Stadbauer TH, Danenberg PV, Ross D and Gibson NW (1992) NAD(P)H : quinone oxidoreductase gene expression in human colon carcinoma cells: characterization of a mutation which modulates DT-diaphorase activity and mitomycin sensitivity. *Cancer Res* **52**: 797–802

Walton MI, Smith PJ and Workman P (1991) The role of NAD(P)H:quinone reductase (EC 1.6.99.2, DT-diaphorase) in the reductive bioactivation of the novel indoloquinone antitumor agent EO9. *Cancer Commun* **3**: 199–206

Workman P (1994) Enzyme-directed bioreductive drug development revisited: a commentary on recent progress and future prospects with emphasis on quinone anticancer agents and quinone metabolizing enzymes, particularly DT-diaphorase. *Oncol Res* **6**: 461–475

© Cancer Research Campaign 1997